# Rare single nucleotide variants in COL5A1 promoter do not play a major role in keratoconus susceptibility associated with rs1536482

**DOI:** 10.1186/s12886-021-02128-6

**Published:** 2021-10-08

**Authors:** Liubov O. Skorodumova, Alexandra V. Belodedova, Elena I. Sharova, Elena S. Zakharova, Liliia N. Iulmetova, Mukharram M. Bikbov, Emin L. Usubov, Olga P. Antonova, Oksana V. Selezneva, Anastasia Levchenko, Olga Yu Fedorenko, Svetlana A. Ivanova, Raul R. Gainetdinov, Boris E. Malyugin

**Affiliations:** 1 Laboratory of Human Molecular Genetics, Federal Research and Clinical Center of Physical-Chemical Medicine of Federal Medical Biological Agency, 1a Malaya Pirogovskaya Ul, Moscow, Russian Federation 119435; 2 Department of Anterior Segment Transplant and Optical Reconstructive Surgery, S. Fyodorov Eye Microsurgery Complex Federal State Institution, 59a Beskudnikovskiy Blv, Moscow, Russian Federation 127486; 3 Department of Surgery of the Cornea and Lens, Ufa Eye Research Institute, Academy of Sciences of the Republic of Bashkortostan, 90 Pushkina Ul, Ufa, Russian Federation 450008; 4 Laboratory for Genomic Research and Computational Biology, Federal Research and Clinical Center of Physical-Chemical Medicine of Federal Medical Biological Agency, 1a Malaya Pirogovskaya Ul, Moscow, Russian Federation 119435; 5 Theodosius Dobzhansky Center for Genome Bioinformatics, Saint Petersburg State University, 7/9 Universitetskaya Nab, Saint Petersburg, 199034 Russia; 6 Laboratory of Molecular Genetics and Biochemistry, Mental Health Research Institute, Tomsk National Research Medical Center, Russian Academy of Sciences, 4 Aleutskaya Ul, Tomsk, 634014 Russia; 7 Division for Testing and Diagnostics, National Research Tomsk Polytechnic University, 30 Lenina Prosp, Tomsk, 634050 Russia; 8 Addiction Psychiatry and Psychotherapy Department, Siberian State Medical University, 2 Moskovsky Trakt, Tomsk, 634055 Russia; 9 Laboratory of Neuroscience and Molecular Pharmacology, Institute of Translational Biomedicine and Saint Petersburg State University Hospital, Saint Petersburg State University, 7/9 Universitetskaya Nab, Saint Petersburg, 199034 Russia

**Keywords:** Keratoconus, Cornea, SNP, GWAS, Genotyping, Promoter, COL5A1, MPDZ, FOXO1

## Abstract

**Background:**

Keratoconus is a chronic degenerative disorder of the cornea characterized by thinning and cone-shaped protrusions. Although genetic factors play a key role in keratoconus development, the etiology is still under investigation. The occurrence of single-nucleotide polymorphisms (SNPs) associated with keratoconus in Russian patients is poorly studied. The purpose of this study was to validate whether three reported keratoconus-associated SNPs (rs1536482 near the *COL5A1* gene, rs2721051 near the *FOXO1* gene, rs1324183 near the *MPDZ* gene) are also actual for a Russian cohort of patients. Additionally, we investigated the *COL5A1* promoter sequence for single-nucleotide variants (SNVs) in a subgroup of keratoconus patients with at least one rs1536482 minor allele (rs1536482+) to assess the role of these SNVs in keratoconus susceptibility associated with rs1536482.

**Methods:**

This case-control study included 150 keratoconus patients and two control groups (main and additional, 205 and 474 participants, respectively). We performed PCR targeting regions flanking SNVs and the *COL5A1* promoter, followed by Sanger sequencing of amplicons. The additional control group was genotyped using an SNP array.

**Results:**

The minor allele frequency was significantly different between the keratoconus and control cohorts (main and combined) for rs1536482, rs2721051, and rs1324183 (*p*-value < 0.05). The rare variants rs1043208782 and rs569248712 were found in the *COL5A1* promoter in two out of 94 rs1536482+ keratoconus patients.

**Conclusion:**

rs1536482, rs2721051, and rs1324183 were associated with keratoconus in a Russian cohort. SNVs in the *COL5A1* promoter do not play a major role in keratoconus susceptibility associated with rs1536482.

## Background

Keratoconus is the most common primary ectatic disorder associated with thinning, stretching, and conical protrusion of the central part of the cornea. The progression of this disease can lead to severe visual impairment due to corneal irregularities and scarring. The disease most often manifests in adolescence and progresses within 10–15 years [1]. Keratotopography- and optical coherence tomography-based approaches have improved the diagnosis of early-stage keratoconus. However, the diagnosis of subclinical keratoconus remains a challenge [2]. The presence of undiagnosed disease in a patient can lead to severe complications after laser refractive interventions, such as the manifestation of reactive keratoconus and its atypical progression [3, 4]. Thus, the development of approaches for the early diagnosis of keratoconus is highly clinically relevant.

Understanding the genetic basis of keratoconus can facilitate its early diagnosis [5]. The genetic nature of keratoconus has been revealed, and many studies have been conducted to search for loci associated with this disease [5–14]. Recently, it was found that several loci are repeatedly associated with keratoconus in large European ancestry cohorts. Among them are loci near the *COL5A1* gene, near the *FOXO1* gene, and near the *MPDZ* gene. Initially, all three loci were discovered to be associated with central corneal thickness in GWASs [15–17]. Then, this association extended to keratoconus cohorts: the association was replicated in candidate gene studies and GWASs [8, 10, 11, 13, 14]. Loci near *FOXO1* and *MPDZ* were also associated with corneal hysteresis and corneal resistance factors [18, 19].

Keratoconus susceptibility loci, even those reported previously, are poorly studied in Russian patients with keratoconus. Patients with keratoconus from Russia were previously genotyped for rs1536482 and rs7044529 in our pilot study on a small sample [20].

This study aimed to investigate the replication of three SNPs (rs1536482 near the *COL5A1* gene, rs2721051 near the *FOXO1* gene, rs1324183 near the *MPDZ* gene) with keratoconus in the largest sample of patients from Russia. Additionally, we investigated rare variants in the *COL5A1* promoter of keratoconus patients with at least one rs1536482 minor allele.

## Methods

### Ethical statements

The Institutional Review Boards of the S. Fyodorov Eye Microsurgery Complex Federal State Institution, the Federal Research and Clinical Center of Physical-Chemical Medicine, and the Ufa Eye Research Institute approved this study. It was conducted in compliance with the tenets of the Declaration of Helsinki. All subjects signed written informed consent forms.

### Keratoconus patients

Clinically affected patients with keratoconus were recruited at the S. Fyodorov Eye Microsurgery Complex Federal State Institution and the Ufa Eye Research Institute. All patients underwent clinical and functional examination, visual acuity testing, refractometry, ophthalmometry, biomicroscopy, and ophthalmoscopy. During slit-lamp examination, surface asphericity and the presence of thinning, Vogt’s striae, Fleischer’s rings, Descemet’s membrane folds, and stromal scarring were evaluated. Keratoconus patient corneas were also assessed using optical coherence tomography Visante OCT (Carl Zeiss; Oberkochen, Germany) and corneal topography TMS-4 (Tomey; Nagoya, Japan). Characteristic keratoconus patterns of corneal topography images (asymmetry, scatter of axes, a difference in keratometry along one axis (irregular astigmatism)), increased surface asymmetry index and surface regularity index values (> 1) along with a central thickness of less than 480 nm, and an inability to correct visual acuity to 1.0 (20/20) were critical for the differential diagnosis of early disease. The keratoconus stage was determined according to the Amsler-Krumeich classification [21]. Patients with at least stage 1 keratoconus were included; patients with subclinical keratoconus were not included in this study. The patients were graded as follows: stage 1, *n* = 9; stage 2, *n* = 39; stage 3, *n* = 85; and stage 4 – *n* = 17. Patients with syndromic keratoconus, in whom keratoconus was only one of the symptoms of a multisystem disease (for example, Down’s syndrome), were excluded. The patients were all of the European descent.

Patients diagnosed with keratoconus were also asked if they would agree to invite first-degree relatives. If they agreed, then their relatives were invited and consulted about participation in the study. After obtaining written informed consent, the relatives were examined in the same way as the keratoconus patients. Participation also meant that blood collection was allowed. For 50 of the patients with keratoconus, the material was also obtained from relatives. The relatives’ DNA was used only for the cosegregation analysis of variants found in the *COL5A1* promoter.

### Main control group

Participants in the main control group (n = 205) were evaluated at the S. Fyodorov Eye Microsurgery Complex Federal State Institution and the Ufa Eye Research Institute. Medical history collection and a thorough ophthalmic examination were carried out for all potential participants. Patients with keratoconus, glaucoma, medium- to high-degree myopia, or retinal or corneal dystrophy were not included. Acceptable ocular diseases included mild myopia, pseudoexfoliation syndrome, and senile (age-related) cataracts. No subjects with a medical history of early-onset cataracts were included, as early-onset anterior polar cataracts are associated with keratoconus [22]. Participants from the control group were of European descent.

### Additional control group

A control group of 503 participants, described by Levchenko A. et al., was included as an additional control group [23]. Briefly, control participants were recruited as volunteers from university teaching personnel and students, employees of local medical and emergency service institutions, and at-large community members in Tomsk, Kemerovo, and Novosibirsk (245 (48.71%) males, mean age 31.2 (SD 9.8); 258 (51.29%) females, mean age 34.0 (SD 15.4); total mean age 32.6 (SD 13.0)). The participants did not undergo an ophthalmologic examination, but since the prevalence of keratoconus in the general population is low, this is unlikely to have an effect on the results.

### Blood collection and DNA extraction

Venous blood (4–6 mL) was collected from each participant in Vacutainer tubes with EDTA (Becton, Dickinson and Company; New Jersey, USA). The samples were stored at − 20 °C until genetic study. DNA was isolated from blood samples with the Gentra Puregene Blood Kit (Qiagen; Hilden, Germany) according to the manufacturer’s protocol. DNA was resuspended in low-TE buffer at a final concentration of 10 ng/μl.

### Genotyping of candidate SNVs

SNV genotyping was carried out by Sanger sequencing. The primer sequences for rs1324183 and rs2721051 were obtained from an article by Liskova P. et al. (Table 1) [11]. Other primers were designed with Primer-BLAST (provided in the public domain by the National Center for Biotechnology Information, Bethesda, MD, USA; Table 1). The applied PCR conditions are described below. ABI sequencing files were analyzed in UniPro UGENE (v37.0) [24].

**Table 1:** Primers Used for Keratoconus-Associated SNV Genotyping

dbSNP identification number	Forward primer sequence 5′ > 3’	Reverse primer sequence 5′ > 3’
rs1536482	AGGTCCCTTGAGCCCTTTTA	TCACCTGAGCCTCCTCATCG
rs2721051	CCAAGGTTAACCGAAGTCCA^a^	AAGGGAAGAGGCAAATGTGA^a^
rs1324183	TATTGATCCACAGCCAGCAG^a^	AAGCGCTTCTAAAAGCCAATC^a^
	ACAGCCAGCAGGAAGAGAACAT	ACAGTGACTTCCTCAGACTGGC

^a^ Primers from Liskova P. et al. [11]

### *COL5A1* gene promoter sequencing

To evaluate *COL5A1* promoter variants, we sequenced the NC_000009.12:134,641,188–134,642,551 region (between 1000 nucleotides upstream of the *COL5A1* transcription start site and the 3′-side of the CpG island in the first exon) [25]. This region also included the promoter-like signature EH38E2735243 [26]. Primers for the promoter region were designed using Primer-BLAST (Table 2). The PCR conditions and the sequencing procedure are described below. Sanger chromatograms were processed with variant analysis software from the cloud dashboard of ThermoFisher Scientific (Waltham, Massachusetts, USA), Unipro UGENE, and Nucleotide BLAST software (provided in the public domain by the National Center for Biotechnology Information, Bethesda, MD, USA).

**Table 2:** Primers Used for COL5A1 Gene Promoter Sequencing

Primer pair name	Genomic coordinate GRCh38	Forward primer sequence 5′ > 3’	Reverse primer sequence 5′ > 3’
COL5A1-F/R4	chr9:134641407–134,641,983	CGAAGTGATCAAACCTCGGG	GCTCGACTTTGGCCCGC
COL5A1-F/R6	chr9:134640893–134,641,555	GAGAGACGCCCACCTACCAC	TTCTCTGAGAGCCGGTAGGC
COL5A1-F/R7	chr9:134641693–134,642,213	CCGGGCTCTGATTTGCTG	GCTTTCCAGCGGGTATGGA
COL5A1-F/R8	chr9:134642371–134,643,040	CTTCGCCCGCAGAACTTTTC	TCATCCCAACCACTCACAGC
COL5A1-F/R9	chr9:134642076–134,642,671	CTAAAGTGGTGCGGTCCCTG	GTTCTGCTGTAGGGCTGTGA

### PCR and sanger sequencing

For the amplification of target sequences, we used the Gene Pak PCR MasterMix Core Kit (IsoGene Lab. Ltd.; Moscow, Russia) under the following conditions: reaction volume, 20 μl; DNA input, 50 ng; and final concentration for each primer, 0.3 nM. Sanger sequencing was carried out according to the manufacturer’s protocol with the BigDye Terminator v3.1 Kit (Thermo Fisher Scientific) on an ABI Prism 3730XL Genetic Analyzer (Applied Biosystems Inc.; Waltham, Massachusetts, USA).

### SNP array analysis

An additional control group was genotyped by Levchenko A. et al. using the iScan System and the Infinium Global Screening Array-24 v1.0 BeadChip (Illumina; San Diego, California, USA) [23]. We converted the SNP array data to PLINK format to perform basic quality control steps using PLINK (v1.9 and v2.0) [27]. We established a quality filter with a threshold of 10% for the SNP missing rate. All individuals exhibiting more than 7% missingness, outlying heterozygosity, 2nd-degree or closer relationships were omitted from the calculation. A total of 474 samples and 640,014 SNPs with a 99.9% genotyping call rate passed all QC requirements and were selected for further consideration.

Genotypes were initially presented in the SNP array data for only rs1536482 and rs2721051, which could thus be directly analyzed by PLINK. The MAF and minor homozygote frequency (MHF) for rs1324183 SNV were estimated after the imputation procedure. Phasing and imputation using the 1000 Genomes Phase III reference panel were conducted with SHAPEIT (v2 (r904)) and IMPUTE2 (v2.3.2), respectively [28–30].

### Statistics

We assessed the differences in allele frequencies between the control and keratoconus groups by using a two-tailed Fisher’s exact test in Prism 7 (GraphPad Software Inc.; San Diego, California, USA) according to a threshold of *p* < 0.05. The Hardy-Weinberg equilibrium of the genotypes in the keratoconus and control groups was tested by the chi-squared test according to a *p*-value = 0.05. Power was calculated in G*Power software (v3.1) [31].

### Meta-analysis

We included studies conducted in European-Ancestry cohorts of keratoconus patients [6–8, 10, 11, 14]. Meta-analysis was conducted with the Meta package (v4.9–6) of R statistical software [32, 33]. We used a random-effect model with the Hartung-Knapp-Sidik-Jonkman estimator for τ^2^ for effect size pooling, which is the preferred method for a small number of unequally sized studies [34]. Heterogeneity was assessed using the *I*^2^ measure [35].

### Availability of data and materials

The datasets used during the current study and ABI trace files are available from the corresponding author on reasonable request.

## Results

### Study design

We included 150 unrelated patients with sporadic (*n* = 130) and familial keratoconus (*n* = 20). The main control group counted 205 unaffected control subjects from the European part of Russia (Table 3). The proportions of males and females in the keratoconus and main control groups did not differ significantly (*p*-value > 0.05). The mean age of the subjects in the main control group was greater than that in the keratoconus group to minimize the number of participants with undiagnosed keratoconus.

**Table 3:** Demographic Characteristics of the Keratoconus and Control Groups

Characteristic		Keratoconus patients (*n* = 150)		Main control group (*n* = 205)	
		Males	Females	Males	Females
Number (%)		104 (69.3%)	46 (30.7%)	129 (62.9%)	76 (37.1%)
Age	Mean age [SD]	31.7 [9.9]	33.9 [11.4]	59.6 [10.7]	67.0 [10.3]
		32.4 [10.4]		62.3 [11.1]	

Due to the lack of genome projects including large cohorts of people from Russia to strengthen the results, we included an additional control group of 474 participants genotyped using an SNP array [23]. This cohort was first used as a control population for our main control group. Then, we combined the data from the main and additional groups and recalculated the results.

### Genotyping of candidate SNVs

All 150 patients with keratoconus and 205 participants from the main control group were genotyped for three SNPs: rs1536482, rs2721051, and rs1324183. Data on the occurrence of each genotype in the keratoconus and control groups are presented in Table 4. Hardy-Weinberg equilibrium was confirmed for all variants except for rs1324183 in the additional control group. We considered this deviation to be a small-sample-size effect.

**Table 4:** Genotypes of the Keratoconus and Control Groups Determined in This Study

dbSNP identification number and related gene	Genotype	Number of subjects with the corresponding genotype, (%)			Chi-squared test p-value		
		Keratoconus group	Main control group	Additional control group	Keratoconus group	Main control group	Additional control group
rs1536482 (near *COL5A1* gene)	A/A	24 (16.0)	16 (7.8)	42 (8.4)	0.7860	0.9580	0.5653
	G/A	70 (46.7)	82 (40.0)	207 (41.2)			
	G/G	56 (37.3)	107 (52.2)	225 (44.8)			
rs2721051 (near *FOXO1* gene)	T/T	2 (1.3)	1 (0.5)	4 (0.8)	0.8824	0.7061	0.3739
	C/T	33 (22.0)	22 (10.7)	63 (12.5)			
	C/C	115 (76.7)	182 (88.8)	407 (80.9)			
rs1324183 (near the *MPDZ* gene)	A/A	0 (0.0)	11 (5.4)	17 (3.7)^a^	0.1795	0.2216	0.0480
	A/C	23 (15.3)	60 (29.3)	111 (24.0)^a^			
	C/C	127 (84.7)	134 (65.3)	334 (72.3)^a^			

^a^ imputed frequency

### Minor allele frequency of candidate SNVs in the additional and Main control groups

Two candidate SNPs, rs1536482 and rs2721051, were directly genotyped from the SNP array data for the additional control group. The IMPUTE2 *info* metric, which estimates the accuracy of imputation, showed a high confidence score for rs1324183. Therefore, we used the imputed rs1324183 genotypes for the additional control group in the analysis. Genotyping data are presented in Table 4, and the MAF data are presented in Table 5. The MAFs in the main and additional control groups did not differ significantly (*p*-value < 0.05 for Fisher’s exact test). Therefore, we merged the two control groups to increase the analysis power.

**Table 5:** Minor Allele Frequency in Keratoconus Patients and Control Groups

dbSNP identi-fication number	Minor allele frequency				Keratoconus vs. main control group		Keratoconus vs. combined control group	
	Keratoconus group	Main control group	Additional control group	Combined control group	p-value	OR	p-value	OR
rs1536482	0.393	0.278	0.307	0.298	0.0016	1.68	0.0016	1.53
rs2721051	0.147	0.059	0.075	0.070	0.0001	2.76	4.60^−05^	2.29
rs1324183	0.290	0.200	0.157^a^	0.169	0.0058	1.63	4.00^−06^	2.01

^a^ imputed frequency

### Minor allele frequency assessment between the keratoconus and control groups

The minor allele distribution was significantly different (*p*-value < 0.05) between the keratoconus and main control groups for all three SNPs: rs1536482 near the *COL5A*1 gene, rs2721051 near the *FOXO1* gene, and the rs1324183 minor allele near the *MPDZ* gene (Table 5). The MAF was also significantly different (p-value < 0.05) between the keratoconus and combined control groups for rs1536482, rs2721051, and rs1324183 (Table 5).

The maximum post hoc power of 89.26% was achieved in the combined control group for rs1324183. rs2721051 in the combined control group showed an acceptable power of 80.04%. rs1536482 showed moderate power of 59.02% in the combined control group.

### Minor allele frequencies among Russian cohort and other European-ancestry cohorts in keratoconus studies

**Fig. 1:**
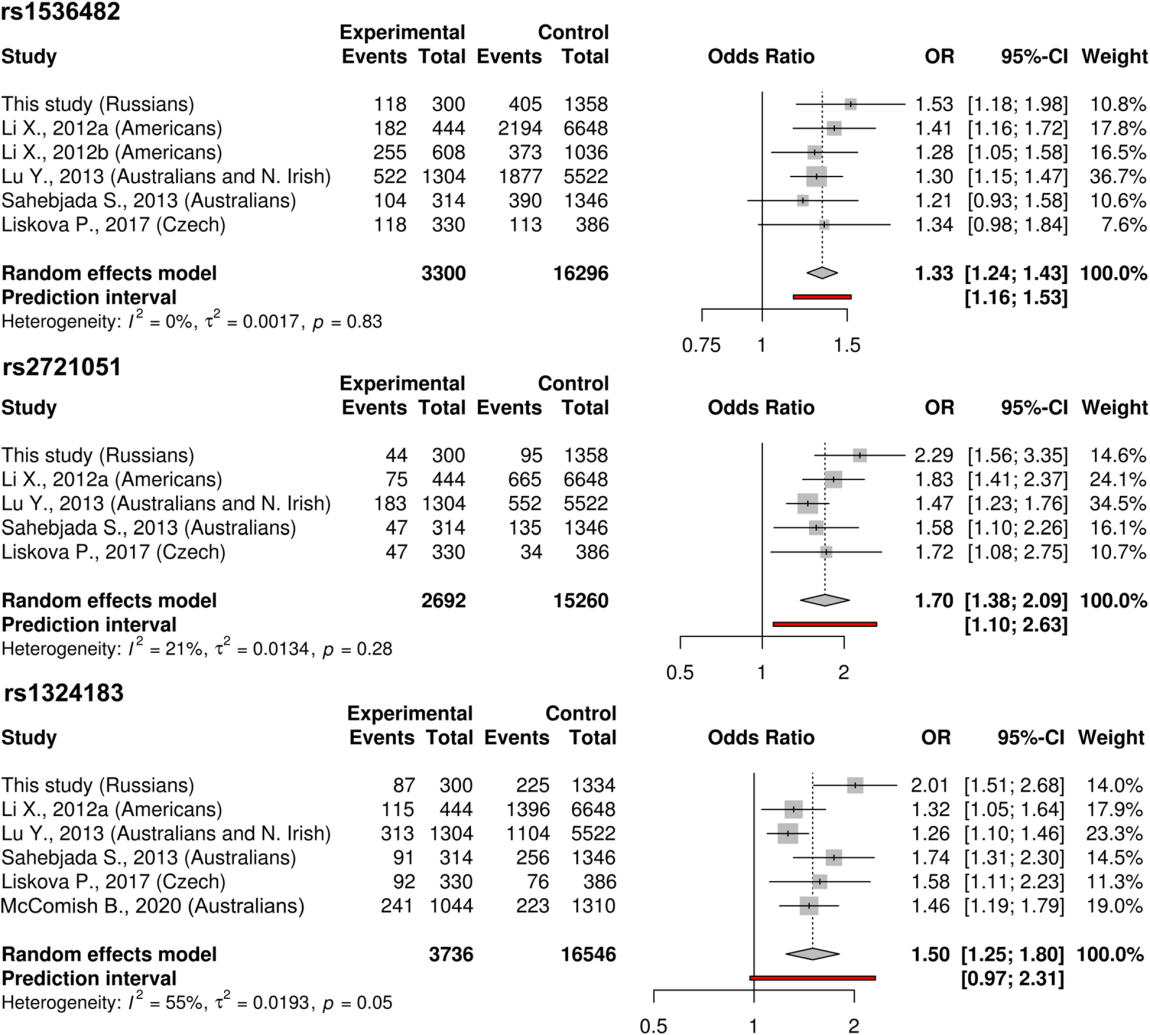
Meta-analyses of the rs1536482, rs2721051, and rs1324183 variants from keratoconus studies. **a** – discovery cohort; **b** – replication cohort

To compare the results for our cohort with those from other reported studies on keratoconus-associated loci, we carried out a meta-analysis of rs1536482, rs2721051, and rs1324183 using the genotyping data from our keratoconus group and combined control group. The results of this analysis are provided in forest plots in Fig. 1. For rs1536482 and rs2721051, pooled effect sizes were homogeneous across studies (*I*^2^  ≤30%). For rs1324183, the *I*^2^ value for heterogeneity was moderate – 55%.

### SNVs in the *COL5A1* promoter

The *COL5A1* promoter (NC_000009.12: 134641188–134,642,551) was sequenced in a subgroup of keratoconus patients with at least one rs1536482 minor allele (*n* = 94). We detected three SNVs with European population frequencies ≤1% according to the ALFA project: rs1043208782, rs569248712, and rs548525119 (Table 6) [36]. Cosegregation analysis was available for rs569248712 (one family) and rs548525119 (two families). Although our group was enriched with subjects harboring the rs548525119 minor allele, we did not observe segregation in either family. rs569248712 cosegregated with the phenotype.

**Table 6:** Rare variants in the COL5A1 promoter in keratoconus patients with the rs1536482 minor allele/es

dbSNP identification number	Number of probands with variant	Sequence annotation	MAF ALFA European	Genotypes	Cosegregation analysis available	Number of cosegregation analyses	Segregation
rs1043208782	1	intron	T = 0.00132	hetero-zygote	no	N/A	N/A
rs569248712	1	5-UTR	A = 0.00399	hetero-zygote	yes	1	yes
rs548525119	3	exon p.Pro21Ser	T = 0.00021	2 homo-zygotes/1 hetero-zygote	yes	2	2 no

## Discussion

Here, we present the results of the genotyping of keratoconus-associated SNPs in a Russian cohort of 829 subjects. We found that three selected variants, rs1536482, rs2721051, and rs1324183, had a significant difference in MAFs between the keratoconus and control groups. Additionally, we compared the pooled effect sizes of rs1536482, rs2721051, and rs1324183 from reported keratoconus association studies of European ancestry cohorts to characterize the placement of the Russian keratoconus cohort. We found that rs1536482 and rs2721051 had homogeneous effect sizes in European-ancestry keratoconus cohorts, including the Russian cohort. This may indicate that these populations exhibit a consistent fraction of patients harboring causal variants in linkage disequilibrium blocks containing minor alleles of these variants. rs1324183 showed a higher OR in the Russian keratoconus cohort than in other European ancestry cohorts. This result reflected the low MHFs (5.4 and 3.4%) in the control groups, which may be a specific feature of the Russian population. This finding needs further investigation in larger samples.

SNPs rs1536482, rs2721051, and rs1324183 are located in the intergenic space, so their direct association with nearby genes is uncertain. Nevertheless, we present some data on the possible role of COL5A1 in the pathogenesis of keratoconus. The nearest gene to rs1536482 with a known function is *COL5A1*, which encodes the collagen type V alpha 1 chain. Collagen V type is a regulatory and structural component of collagen fibrils. In the cornea, it assembles with collagen type I into heterotypic collagen fibrils. Mutations in the *COL5A1* gene are the most common cause of Ehlers-Danlos syndrome [37]. In patients with Ehlers-Danlos syndrome, mutations in the *COL5A1* gene lead to a decrease in corneal thickness and the density of collagen fibrils, similar to the changes observed in keratoconus [38, 39]. The keratoconus patients in our sample did not have Ehlers-Danlos syndrome, so we did not expect them to harbor pathogenic variants in the protein-coding sequence of *COL5A1*. Additionally, it was previously found that patients with keratoconus do not show enrichment in rare potentially pathogenic variants in protein-coding sequences, and only intron variants of unknown significance were found [40, 41]. Therefore, we considered the possibility that potentially pathogenic variants may be located in the *COL5A1* promoter. We sequenced the *COL5A1* promoter in a subgroup of keratoconus patients who had at least one rs1536482 minor allele. We identified three rare SNVs. Among these SNVs, rs548525119 did not cosegregate in relatives. The involvement of rs1043208782 and rs569248712 in keratoconus pathogenesis needs further investigation. These SNVs were present in a small proportion of keratoconus patients (two out of 94). Therefore, we conclude that rare SNVs in the *COL5A1* promoter do not play a major role in keratoconus susceptibility associated with rs1536482. This is consistent with our results showing that the presence of the rs1536482 minor allele is not associated with the *COL5A1* gene expression level [20].

The study is limited by its relatively modest sample size. However, the sample size examined in our study was comparable to those in other candidate gene studies of keratoconus [10, 11].

## Conclusions

We found that rs1536482 near the *COL5A1* gene, rs2721051 near the *FOXO1* gene, and rs1324183 near the *MPDZ* gene were significantly associated with keratoconus in a Russian cohort. We found two rare variants in the COL5A1 promoter, one of which, rs569248712, cosegregated with the phenotype in the analyzed family.

## Data Availability

The datasets used during the current study and ABI trace files are available from the corresponding author on reasonable request.

## Abbreviations

ABI: Applied Biosystems Inc. sequencer trace file format
EDTA: Ethylenediaminetetraacetic acid
GWAS: Genome-wide association study
MAF: Major allele frequency
MHF: Minor homozygote frequency
OR: Odds ratio
PCR: Polymerase chain reaction
SD: Standard deviation
SNP: Single nucleotide polymorphism
SNV: Single nucleotide variant
